# Learning from the COVID-19 pandemic among migrants: An innovative, system-level, interdisciplinary approach is needed to improve public health

**DOI:** 10.1177/14034948211019795

**Published:** 2021-05-31

**Authors:** Esperanza Diaz, Svenn-Eirik Mamelund, Jarle Eid, Henriette Sinding Aasen, Oddvar Martin Kaarbøe, Rebecca Jane Cox Brokstad, Siri Gloppen, Anders Beyer, Bernadette Nirmal Kumar

**Affiliations:** 1 Pandemic Centre, University of Bergen, Norway; 2 Department for Global Public Health and Primary Care, Faculty of Medicine, University of Bergen, Norway; 3 Unit for Migration and Health, Norwegian Institute of Public Health (FHI), Norway; 4 Centre for Research on Pandemics, OsloMet, Norway; 5 Centre for Crisis Psychology, University of Bergen, Norway; 6 Faculty of Law, University of Bergen, Norway; 7 Faculty of Social Sciences, University of Bergen, Norway; 8 Faculty of Medicine, University of Bergen, Norway; 9 Department of Comparative Politics, Faculty of Social Sciences, University of Bergen, Norway; 10 Faculty of Fine Art, Music and Design, University of Bergen, Norway

**Keywords:** Migrants, pandemic, COVID-19, paradigm, interdisciplinarity

## Abstract

The effects of the COVID-19 pandemic are amplified among socially vulnerable groups, including international migrants, in terms of both disease transmission and outcomes and the consequences of mitigation measures. Migrants are overrepresented in COVID-19 laboratory-confirmed cases, hospital admissions, intensive care treatment and death statistics in all countries with available data. A syndemic approach has been suggested to understand the excess burden in vulnerable populations. However, this has not stopped the unequal burden of disease in Norway. Initially, the disease was mainly imported by Norwegians returning from skiing holidays in the Alps, and the prevalence of infection among migrants in Norway, defined as people born abroad to foreign parents, was low. Later, confirmed cases in migrants increased and have remained stable at 35–50% – more than twice the proportion of the migrant population (15%). To change this pattern, we need to understand the complex mechanisms underlying inequities in health and their relative and multiplying impacts on disease inequalities and to test the effect of counterfactual policies in order to reduce inequalities in disease burden. Yet, the current paradigm in the field of migration and health research, that is, the theories, research methods and explanatory models commonly applied, fail to fully understand the differences in health outcomes between international migrants and the host population. Here, we use the Norwegian situation as a case to explain the need for an innovative, system-level, interdisciplinary approach at a global level.

The pandemic’s effects are amplified among socially vulnerable groups, including international migrants, in terms of both disease transmission and outcomes and the consequences of mitigation measures. Migrants are overrepresented in COVID-19 laboratory-confirmed cases, hospital admissions, intensive care treatment and death statistics in all countries with available data [1–3]. A syndemic approach has been suggested to understand the excess burden in vulnerable populations [4]. However, this has not stopped the unequal burden of disease in Norway, as seen in Figure 1. Initially, the disease was mainly imported by Norwegians returning from skiing holidays in the Alps, and the prevalence of infection among migrants in Norway, defined as people born abroad to foreign parents, was low. Later, confirmed cases in migrants increased and have remained stable at 35–50% – more than twice the proportion of the migrant population (15%). To change this pattern, we need to understand the complex mechanisms underlying inequities in health and their relative and multiplying impacts on disease inequalities and to test the effect of counterfactual policies in order to reduce inequalities in disease burden [5]. Yet, the current paradigm in the field of migration and health research, that is, the theories, research methods and explanatory models commonly applied, fail to fully understand differences in health outcomes between international migrants and the host population. Here, we use the Norwegian situation as a case to explain the need for an innovative, system-level, interdisciplinary approach at a global level.

**Figure 1.**
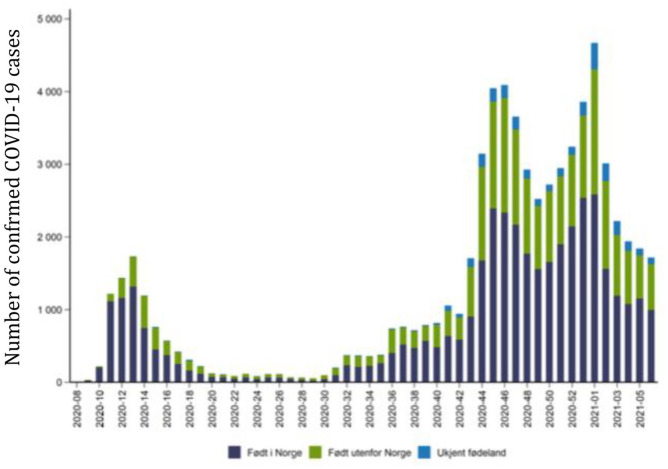
Number of COVID-19 cases in people born in Norway (blue), born abroad (green) and unknown (light blue), 17 February 2020–14 February 2021. Source: FHI.

A lack of information and cultural differences are often cited as the main causes of the disproportionate disease burden among migrants [6]. However, despite targeted information campaigns during the pandemic, many migrant groups in Norway reported that the recommended measures did not specifically address their everyday life challenges [7]. The fact that the migrant groups hardest hit in the first phase of the pandemic, defined from the start of restrictive measures until the beginning of a gradual opening of society in May 2020, also featured at the top of the pandemic statistics in the second and third waves [8] indicates that mechanisms other than health knowledge are important. In Sweden, lower socio-economic status (SES) can explain half of the increased COVID-19 incidence among migrants [9]. Evidence from other European countries outside Scandinavia points to the complexity of the situation. In Italy, structural factors contributed to delayed diagnosis of COVID-19 disease, especially for women from low-income countries [3]. In the UK, deprivation and pre-existing clinical risk factors explained very little of the increased risk of in-hospital death from COVID-19 among Asian and black people [10]. In most countries, undocumented migrants face serious problems in accessing adequate protection and care [11]. Despite emerging empirical evidence, we still need a comprehensive model to understand the interactions and outcomes at the individual and structural levels in order to explain and alleviate the extra burden of COVID-19 in the migrant population [12].

The collateral effects of the pandemic due to the economic downturn, social isolation and movement restrictions are also unequally affecting those in the lowest strata of society [13], dubbed the ‘double burden of COVID-19’ [14]. Psychological distress [15], food insecurity [16] or unemployment in the first period of the pandemic [17] are examples of how migrants have been hit harder both by the pandemic and by the measures adopted to stop the disease. However, little is still known about the mechanisms and long-term effects of such structural factors on pandemic outcomes and whether they can be explained by inequalities in exposure, underlying susceptibility and/or access to care/health knowledge.

The pandemic situation is unique in that all members of society must confront an invisible threat: potential infected people in local communities, workplaces and among family and friends. The social stigma associated with the pandemic causes uncertainty and anxiety. Rumours and conspiracy theories about COVID-19 have spread rapidly, fuelling mistrust of governments and minority groups. Lack of trust has been highlighted as a key factor in explaining poor adherence to hygiene recommendations among migrants [1]. In their attempt to build trust, several European governments supported migrant communities to deliver information to their peers. However, the encouragement of migrant ‘influencers’ to promote health within their own groups has been found to divide rather than unite society [18].

Unfortunately, the future does not promise significant improvements for migrants. Our hopes rely on vaccination campaigns, but research suggests that migrants generally experience a higher vaccine preventable disease burden and lower immunisation rates [19]. In Norway, a national survey from late 2020 showed that 73% intended to be vaccinated, but that the elderly, men, those with no or a weak connection to the labour market, recipients of social welfare, migrants from Asia and Africa and migrants with little contact with the majority of the population had lower intentions to be immunised [20]. If no specific measures are adopted, migrants will probably be vaccinated against COVID-19 to a lesser degree than the majority of the population. In addition, the priority for vaccination is mainly age based, which overlooks that among certain migrant groups, the severity of the virus infection seems to occur at a younger age [9,21]. Criteria developed based on typical risk factors in the general population may miss factors relevant to these groups. International human rights law requires non-discrimination and equal access to necessary and adequate services, which includes access to vaccination programmes on equal terms. Thus, priority settings and legal factors need to be addressed.

During the pandemic, workers have often been classified in terms of their participation in jobs necessary to maintain the continuity of operations of essential critical infrastructure sectors. Globally, essential workers and their families, many of whom are migrants, have borne the brunt of the COVID-19 pandemic [22]. Pandemics and outbreaks create fear, which increase racism and xenophobia [23]. Racism has been acknowledged as a probable cause of the excess burden of COVID-19 disease among migrants and ethnic minorities [6,23]. Individuals who have suffered discrimination become increasingly vulnerable, and those who are infected find it harder and slower to recover [24]. Furthermore, many working migrants are employed precariously and are thus ineligible for sick leave, social security or COVID-19 special payments [25]. The government’s decision to give Norwegian employers of international workers the possibility of being exempted from the quarantine rules when entering the country was widely criticised and has now turned to a nearly complete ban on labour migration. However, migrants themselves are often blamed for the import and spread of infections in traditional media and particularly on social media, while there is paucity of evidence-based communication from public authorities accounting for the complex causes of migrants’ disproportional COVID-19 burden. This may in turn contribute to the stigma, misinformation and oversimplification of underlying causes – and to sustained high levels COVID-19 infections among migrants.

Access to care is another cornerstone of equity in health. The Norwegian state is legally obliged to ensure equal and non-discriminatory access to health services for all, but there are two groups of people who are not covered: undocumented migrants and working migrants during the first months of residence in the country. Both groups are entitled to emergency health care, but none of them have the right to enrol with a general practitioner. Although there are no COVID-19 statistics for undocumented migrants, the prevailing high burden of COVID-19 disease among labour migrants makes it reasonable to believe that health-care services are not in line with the legal requirements of non-discrimination. Moreover, in Norway, the migrant background of persons is not registered systematically in health registers or in electronic medical records [4], despite this being recommended by national and international experts [6,7]. This is due to data protection law and privacy considerations. The lack of relevant data constitutes a serious structural barrier to address migrant health properly in a pandemic situation. A systematic registration of migrant backgrounds in health registers could improve preparedness for better health-care provision.

## Need for innovative, system-level interdisciplinarity to disentangle mechanisms of disease among migrants and to improve public health

From the above, it is clear that by studying individual factors from a single discipline, we cannot disentangle the complexity and explain the disparities in health between migrants and native-born Norwegians [26]. Theoretical models for migrant health outcomes tend to over-rely on individual explanations, with an emphasis on the personal cultural background, which obscures the influence of structural factors impacting migrant health disparities, such as policies, regulations, work opportunities, information in health registers, stigma or poor socio-economic conditions. Non-medical academic environments focus more often on racism and the impact of national migrant policies as reasons for inequalities in health. Acknowledging the importance of including all the previous factors to advance the research field of migration and health, we need new perspectives that address how multiple structural dimensions of systemic discrimination in several life areas intersect to impact health outcomes [6]. Furthermore, the biomedical paradigm in itself represents a structural barrier, particularly in a pandemic, preventing doctors, public health planners, emergency preparedness institutions and governments from think about non-medical vulnerabilities and indications for interventions [27].

COVID-19 is a complex, ‘wicked problem’ [28] that has to be confronted by an interdisciplinary collaborative approach. While the evidence points to *what* has gone wrong, we need to understand *why*. In that quest for answers, our theories need to be tried and tested. In order to take this forward, the University of Bergen (UiB) recently established the Pandemic Centre. This interdisciplinary centre represents a cross-faculty effort at the UiB to develop large-scale, interdisciplinary and user-inclusive approaches jointly in order to disentangle the complex causes of increased COVID-19 burden with the ultimate aim of improved public health. The core idea is that the pandemic will highlight and profoundly sharpen the disparities in public health – but also that it can reveal the underlying drivers. This is particularly the case for people with international migrant backgrounds, including both documented and undocumented migrants with all causes of migration, where the effects of social, economic, cultural and legal structures interact with health outcomes in complex ways, impacting all areas of life [29]. While current research mainly studies medical risk factors, we propose a radically transdisciplinary perspective, including the individual and system levels, for the study of risk factors and their interaction, thus producing new information to inform future pandemic preparedness better and to contribute to fewer inequalities and better health for society at large. With a shared research agenda and research questions from different theoretical perspectives and research traditions, we will produce new information that will undergo an additional burden of proof, also put under the scrutiny of disciplines other than one’s own [30]. We will also include users, civil society and policymakers as collaborators in the research to ensure that the evidence produced is actionable and suited to assist in decreasing health inequalities, fighting stereotypes and discrimination and improving pandemic preparedness. In this way, we will advance our understanding of the broader societal and human implications from the COVID-19 pandemic. Ultimately, we hope to be able to contribute to shifting the agenda of migrant research from a current conflict-oriented approach into a more sustainable, constructive and solution-oriented research and thereby contribute to better health for all.
